# High-Risk Bleeding in Acute Myeloid Leukemia: Clinical Presentation, Laboratory Markers, and Mortality Trends

**DOI:** 10.1177/10760296251405424

**Published:** 2025-12-10

**Authors:** Mohammed Abdulgayoom, Yousef Al-Asa'd, Ahmed Ali, Abdulrahman F Al-Mashdali, Hawra Shawaylia, Dina Sameh Soliman, Deena Mudawi, Feryal Abbas, Omran Almokdad, Anil Ellahie, Honar Cherif, Shehab Mohamed

**Affiliations:** 1 Department of Hematology, 62850 National Center for Cancer Care and Research, HMC, Doha, Qatar; 2 Department of Internal Medicine, 62849 Hamad General Hospital, HMC, Doha, Qatar; 3 Department of Laboratory Medicine and Pathology, 36977 National Center for Cancer Care and Research, Doha, Qatar; 4 Department of Clinical Imaging, Interventional Radiology Section, 36977 Hamad General Hospital, HMC, Doha, Qatar

**Keywords:** acute myeloid leukemia, AML, bleeding, hemorrhage, intracranial hemorrhage

## Abstract

**Background:**

Severe bleeding is a major complication in acute myeloid leukemia (AML), contributing to early mortality. While thrombocytopenia and coagulopathy are known risk factors, the clinical and laboratory features surrounding haemorrhagic events in real-world AML cohorts require further elucidation.

**Objectives:**

To assess the frequency, sites, clinical features, laboratory findings, and outcomes of Grade ≥3 bleeding in AML patients.

**Methods:**

This retrospective study reviewed 214 AML patients treated between 2012 and 2021 at a tertiary centre in Qatar. Patients with WHO Grade ≥3 bleeding were analysed for clinical, laboratory, cytogenetic, and outcome data.

**Results:**

Thirty-five patients (16.3%) had Grade ≥3 bleeding, most commonly intracranial (86%), presenting with neurological symptoms (80%). Events occurred mainly during or soon after induction chemotherapy (40%). Median haemoglobin and platelet count at bleeding were 7.1 g/dL and 11 × 10^9^/L. Most cases (94%) were managed medically; 6% required neurosurgery. Thirty-day mortality was 41.2%, with overall mortality reaching 76.5%. Intracranial hemorrhage and septic shock accounted for most deaths.

**Conclusions:**

Grade ≥3 bleeding, particularly intracranial, is a frequent and deadly event in AML, often arising during induction. Severe cytopenias and coagulopathy are common. Improved risk stratification and supportive care are essential to reduce bleeding-related mortality.

## Introduction

Acute myeloid leukemia (AML) is a clonal hematopoietic malignancy characterised by the proliferation of immature myeloid cells in the bone marrow and peripheral blood, leading to ineffective hematopoiesis and profound cytopenia.^1^ While infections and treatment-related toxicities are widely recognised as major complications during the AML disease course, haemorrhagic events, particularly severe bleeding, represent an equally critical yet often underappreciated contributor to early morbidity and mortality.^2^ Estimates suggest that bleeding of any grade occurs in up to 60% of AML patients during induction therapy, with severe (Grade ≥3) bleeding documented in approximately 5%-20% depending on population and treatment setting.^3–5^ The WHO bleeding scale consists of four grades, with Grade 3 representing severe bleeding requiring transfusion, hospitalization, or intervention, and Grade 4 denoting life-threatening bleeding such as intracranial hemorrhage with hemodynamic instability or major organ dysfunction.^3^

Intracranial hemorrhage, although less common than mucosal or gastrointestinal bleeding, remains the most feared complication due to its acute presentation, management difficulty, and high fatality rate. The risk of severe bleeding in AML is multifactorial, involving thrombocytopenia, coagulopathy, leukostasis, endothelial damage, and chemotherapy-induced marrow suppression.^6^ Furthermore, certain disease subtypes, such as those with *KMT2A* rearrangement, have been associated with heightened bleeding risk, possibly due to more aggressive disease biology or underlying coagulopathy.^7,8^

While acute promyelocytic leukemia (APL) is well known for its distinct coagulopathy and unique haemorrhagic profile, this entity has been intentionally excluded from the current analysis due to its biologically and clinically distinct nature and dedicated therapeutic pathways.^9^ As a result, this study focuses exclusively on non-APL AML patients.

Given the multifactorial nature of bleeding risk and its significant contribution to early mortality, optimal supportive care remains a cornerstone of AML management, as emphasised in recent ELN (2022) and NCCN (2025) guidelines.^10,11^ These guidelines highlight the importance of infection prophylaxis, transfusion support, and coagulopathy management in improving outcomes and reducing early mortality during induction therapy. Although infections and older age remain major contributors to induction mortality, hemorrhage continues to represent one of the leading direct causes of early death in AML.^12,13^ Data on bleeding patterns from non-Western patient populations remain limited, and gaining insight into real-world experiences across diverse healthcare settings is crucial for improving patient outcomes.

This study aimed to characterise the clinical, laboratory, and treatment-related factors associated with Grade ≥3 bleeding in AML patients treated at the National Centre for Cancer Care and Research in Qatar over ten years. By analysing the frequency, timing, location, and outcomes of severe bleeding events in this cohort, we aimed to better characterise the hemorrhagic burden in AML and guide risk stratification and preventive strategies**.**

## Methodology

This study was a retrospective observational analysis conducted at the National Centre for Cancer Care and Research (NCCCR) in Doha, Qatar. The study population consisted of patients newly diagnosed with AML between January 2012 and December 2021. The primary objective was to characterise the clinical, laboratory, and treatment-related factors associated with severe bleeding events in patients diagnosed with AML, excluding those with APL, over ten years.

## Study Population

The study included all adult patients newly diagnosed with non-APL AML at NCCCR between January 1, 2012, and December 31, 2021. A total of 214 patients met the inclusion criteria. Patients with a confirmed diagnosis of APL were excluded due to the distinct biology, clinical behaviour, and bleeding profile of this AML subtype. From the total cohort, patients who developed Grade 3 or higher bleeding events—as defined by the World Health Organisation (WHO) bleeding scale—were identified for detailed analysis.

## Data Collection

Data were extracted through a detailed review of electronic medical records, encompassing demographic variables (including age, sex, and ethnicity), comorbidities such as diabetes mellitus, hypertension, and chronic renal disease, and detailed characteristics of bleeding events. Information collected on bleeding episodes included the anatomical site, mode of diagnosis (eg, CT or MRI imaging), clinical presentation, and their temporal relationship to chemotherapy administration.

Bleeding events were confirmed through clinical documentation and radiologic reports, with emphasis placed on those meeting the WHO Grade ≥3 criteria, which define severe or life-threatening bleeding requiring transfusion, medical intervention, or associated with significant morbidity. To minimise inter-observer bias, all events were retrospectively graded according to the WHO bleeding scale by two independent investigators, and discrepancies were resolved by consensus.

## Laboratory and Genetic Evaluation

Baseline laboratory data included hematological parameters such as haemoglobin (Hb), white blood cell (WBC) count, platelet count, and absolute neutrophil count (ANC). Coagulation profiles were assessed using international normalised ratio (INR), prothrombin time (PT), activated partial thromboplastin time (aPTT), fibrinogen levels, and D-dimer concentrations. Renal and liver function markers including serum creatinine, urea, and albumin were also recorded. Cytogenetic analysis was performed using conventional karyotyping or fluorescence in situ hybridisation (FISH), while molecular profiling included testing for NPM1 and FLT3-ITD mutations when data were available

## Transfusion and Supportive Care Protocols

Platelet transfusion practices at NCCCR followed institutional and international supportive-care guidelines throughout the study period. Prophylactic platelet transfusions were routinely administered when platelet counts fell below 10 × 10⁹/L, or below 20 × 10⁹/L in the presence of fever, active infection, or other bleeding risk factors. For active bleeding or prior to invasive procedures, platelet thresholds were increased to 30-50 × 10⁹/L based on clinical judgement. Red-cell transfusions were provided to maintain haemoglobin above 7-8 g/dL, and plasma or cryoprecipitate was administered for coagulopathy correction as indicated.

## Outcome Measures

The primary outcomes evaluated were the incidence and anatomical distribution of severe (Grade ≥3) bleeding events, along with the clinical presentation and diagnostic findings associated with these episodes. Secondary outcomes included analysis of chemotherapy types and timing relative to bleeding events, strategies used for bleeding management (including supportive care, pharmacologic interventions, and surgical procedures), patient survival at 30, 60, and 90 days following the bleeding event, and the association between laboratory abnormalities and the severity of haemorrhagic complications

## Statistical Analysis

Descriptive statistics were employed to summarize patient characteristics, laboratory values, and bleeding patterns. Continuous variables were reported as means, medians, and ranges, while categorical data were presented as counts and percentages. Proportions are presented with corresponding 95% confidence intervals (CIs) where applicable. Group comparisons were made to identify trends or associations between bleeding severity and clinical/laboratory variables. The impact of various intervention strategies on survival outcomes was explored descriptively.

## Results

During the ten-year study period, 214 patients were newly diagnosed and treated for AML at the National Center for Cancer Care and Research. Of these patients, 35 (16.3%, 95% CI 12.0-21.9) experienced severe bleeding manifestations.

## Demographic Characteristics

Among the 35 patients who experienced Grade ≥3 bleeding, the majority were male (77%, n = 27), while females accounted for 23% (n = 8). Most patients were of Asian ethnicity (63%, n = 22), followed by Arab ethnicity (31%, n = 11). One patient each (3%) was of White and African ethnic origin. The mean age at the time of bleeding was 49.6 years (SD: 16.7 years, range from 24 to 77 years). The age distribution revealed a skew toward middle-aged adults, with 25% of patients under 38 years, 50% under 48 years, and 75% under 63 years (Table 1).

**Table 1.** Demographic Characteristics of AML Patients with Grade ≥3 Bleeding (n = 35).

Category	Characteristic	Value
Overall count	Total number of patients	35
Gender	Male	27 (77%)
	Female	8 (23%)
Ethnicity	Asian	22 (63%)
	Arab	11 (31%)
	White	1 (3%)
	African	1 (3%)
Age	Mean age	49.6 years
	Median age	48 years
	Age range	24-77 years

## Comorbidities

Comorbidity data were available for 31 patients (89%). Hypertension was the most common comorbidity (36%), followed by diabetes mellitus (16%). Only one patient (3%) had chronic renal disease. Overall, 39% of patients had at least one major comorbidity, while 61% had no significant comorbid conditions.

## Disease Characteristics

**Figure 1.**
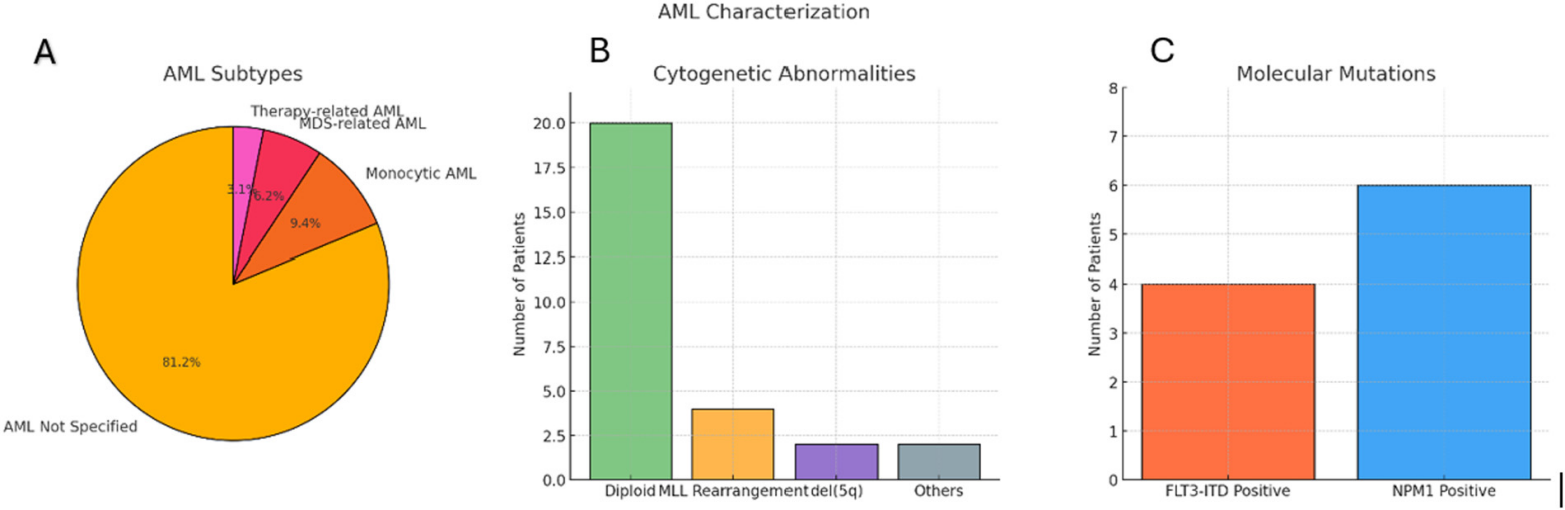
Characterization of AML in 35 patients with grade ≥3 bleeding.

The majority (74%) had (AML) without documented subclassification. AML with monocytic features was present in 9%, AML with myelodysplasia-related changes in 6%, and therapy-related AML in 3%. Cytogenetic analysis most frequently showed a diploid karyotype, though MLL rearrangements, del(5q), and other abnormalities were also observed. Molecular testing identified FLT3-ITD mutations in 4 patients and NPM1 mutations in 6 (Figure 1).

## Bleeding Events

Intracranial hemorrhage was the predominant bleeding site, reported in 86% (n = 30) of cases. This included Intracerebral Hemorrhage ICH (n = 12), Subdural Hemorrhage SDH (n = 7), Subarachnoid hemorrhage SAH (n = 6), cerebellar hemorrhage (n = 2), and combined intracranial bleeding sites (n = 3). Non-intracranial bleeding included gastrointestinal, retroperitoneal, bladder, and soft tissue sites. Most events were radiologically confirmed and often prompted by neurological symptoms.

**Figure 2.**
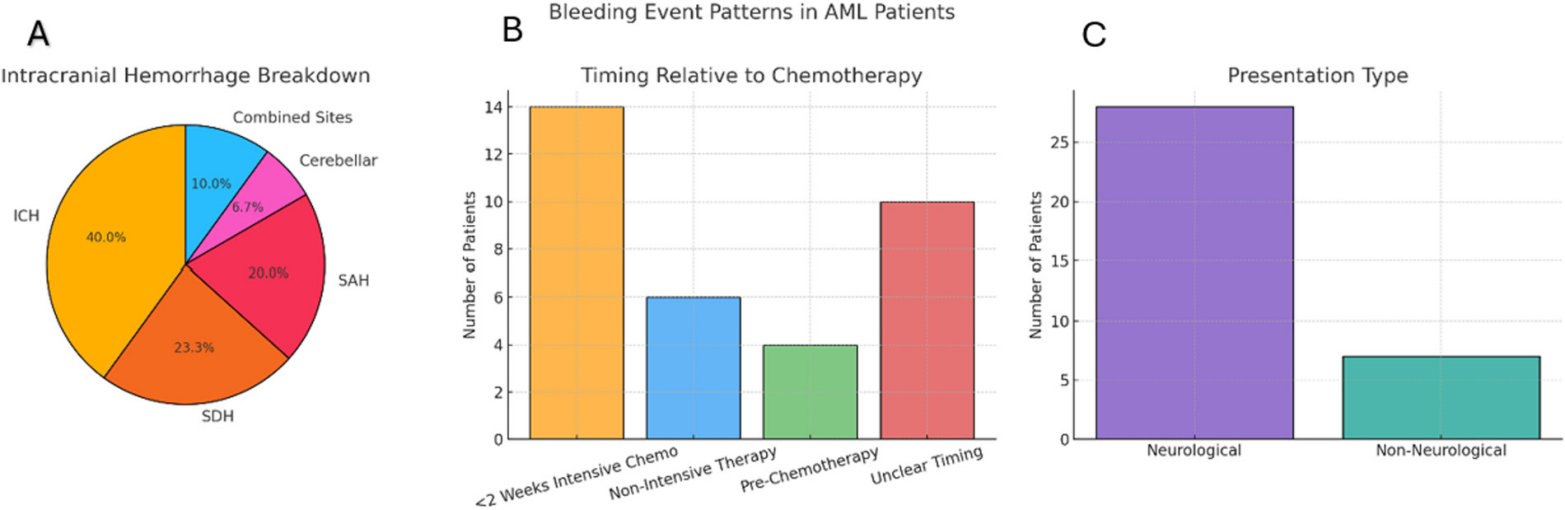
Pattern of sever bleeding events in AML patients.

Timing-wise, 40% (n = 14, 95% CI 25.6-56.4) of events occurred within two weeks of intensive chemotherapy, typically during induction or HiDAC-based regimens. Seventeen per cent (n = 6, 95% CI 8.1-32.7) occurred during non-intensive therapy, such as azacitidine or gilteritinib and 11% (n = 4, 95% CI 4.5-26.0) presented prior to initiation of chemotherapy (All patients had thrombocytopenia, with platelet counts ranging from 14 to 33 × 10⁹/L, and one patient exhibited hyperleukocytosis, with a white blood cell count of 333 × 10⁹/L). In 10 cases (28.6%, 95% CI 16.3-45.1), timing was unclear. Neurological symptoms were present in 80%(n = 28) of cases, such as anisocoria, confusion, loss of consciousness, seizures, or deep coma level (DCL). The remaining seven patients (20%) had non-neurological bleeding presentations, including gastrointestinal, urinary tract, retroperitoneal, and soft tissue bleeding. Two patients were initially asymptomatic and were discovered incidentally during imaging or clinical assessments (Figure 2).

## Lab Findings at Baseline and During the Event

Laboratory investigations conducted at the time of Grade ≥3 bleeding events showed changes compared to baseline measurements. Hemoglobin (Hb) levels at baseline had a mean of 7.9 g/dL and a median of 7.3 g/dL. During bleeding events, the mean was 8.0 g/dL and the median was 7.1 g/dL. The range during bleeding events was 5.5-12.1 g/dL. Platelet counts had a baseline mean of 84.6 × 10⁹/L and a median of 34 × 10⁹/L. During bleeding, the mean platelet count was 62.8 × 10⁹/L and the median was 11 × 10⁹/L. White blood cell (WBC) counts showed a baseline mean of 56.4 × 10⁹/L and a median of 20 × 10⁹/L. During bleeding, the mean was 14.5 × 10⁹/L and the median was 1.3 × 10⁹/L. Absolute neutrophil count (ANC) had a baseline mean of 1.6 × 10⁹/L and a median of 0.8 × 10⁹/L. During bleeding, the mean was 1.1 × 10⁹/L and the median was 0.1 × 10⁹/L.INR at baseline was 1.3 on average and increased to 1.4 during bleeding. Fibrinogen levels, available in a subset of patients, showed a decline from a baseline mean of 3.4 g/L to 2.7 g/L at the time of bleeding. D-dimer levels increased from a baseline mean of 6.8 mg/L FEU to 13.2 mg/L FEU during the event (Table 2).

**Table 2.** Comparison of Hematologic and Coagulation Parameters at Baseline and During Bleeding Events in AML Patients (n = 35).

Parameter	Baseline Median (Range)	Bleeding Event Median (Range)
Hemoglobin (g/dL)	7.3 (3.0-14.7)	7.1 (5.5-12.1)
Platelet Count (×10^9^/L)	34 (6-656)	11 (5-193)
WBC Count (×10^9^/L)	20 (0.7-268)	1.3 (0.1-64.5)
ANC (×10^9^/L)	0.8 (0.0-6.4)	0.1 (0.0-4.1)
INR	1.3 (1.0-1.7)	1.4 (1.0-1.7)
Fibrinogen (g/L)*	3.1 (0.94-6.18)	2.1 (0.94-5.7)
D-dimer (mg/L FEU)*	3.5 (1.1-46.0)	8.2 (1.3-35.2)

*Data available in a subset of patients.

## Interventions

Conservative medical management (including transfusions, hypertonic saline, mannitol and management of other disease concomitant complications), was the primary treatment and was utilized in 33 patients (94%). Two patients (6%) underwent neurosurgical procedures.

## Outcomes

**Figure 3.**
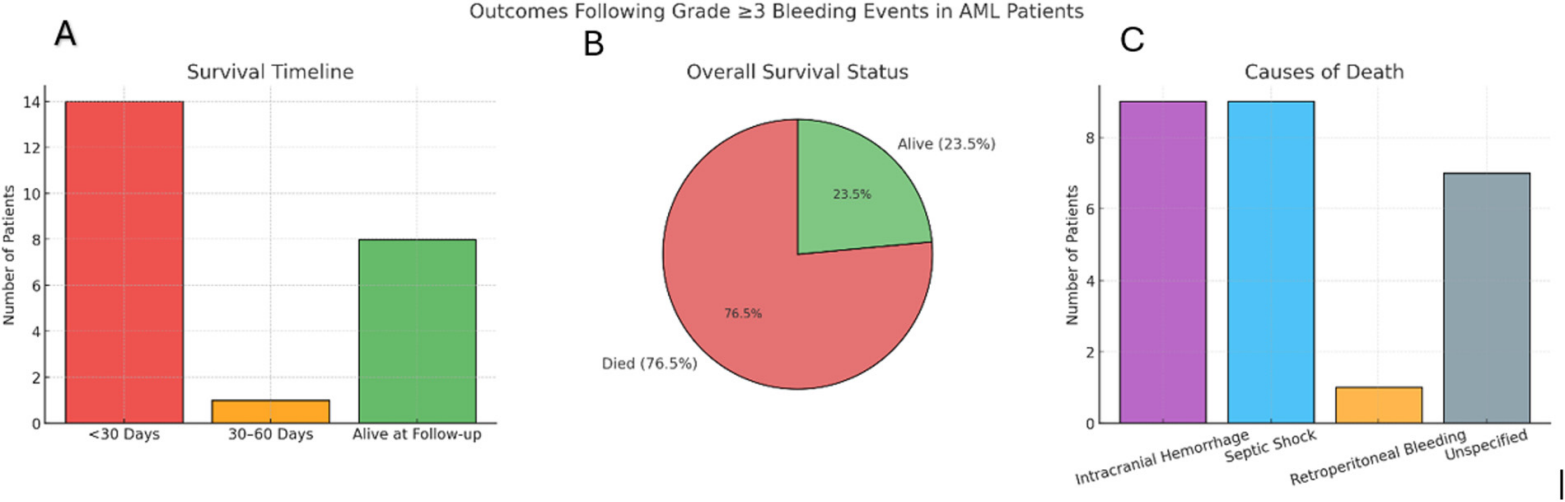
Outcomes following severe bleeding events in AML patients.

Among the 34 patients with documented follow-up, 14 (41.2%, 95% CI 26.4-57.8) died within 30 days of the bleeding event, and one additional patient died between 30 and 60 days. The total mortality rate was 76.5% (n = 26, 95% CI 60.0-87.6), while 23.5% (n = 8, 95% CI 12.4-40.0) were alive at the time of last follow-up (median follow-up duration 5 years). The most common causes of death were intracranial hemorrhage and septic shock, each accounting for 9/26 (34.6%, 95% CI 19.4-53.8). One patient (3.8%, 95% CI 0.7-18.9) died from retroperitoneal hemorrhage, while 7/26 (26.9%, 95% CI 13.7-46.1) were due to unspecified causes (Figure 3). Survivors were more likely to have experienced less extensive bleeding or to have received timely medical or surgical intervention. Survival was notably higher among patients who bled after completing intensive chemotherapy or during hematologic recovery, compared to those who bled during induction.

## Discussion

This retrospective study highlights the critical burden of severe bleeding complications in patients with AML, revealing a complex interplay of disease biology, treatment-related factors, and clinical vulnerability. Among 214 newly diagnosed AML patients treated at our center over a ten-year period, 16.3% experienced Grade ≥3 bleeding, with intracranial hemorrhage being the predominant and most devastating manifestation. This aligns with previous studies that have consistently identified central nervous system bleeding as a major source of morbidity and early mortality in AML^13^ and is comparable to the 5%-20% incidence reported in other international series.^3–5^

Demographically, our cohort reflected the ethnic composition of Qatar's patient population, with a predominance of Asian and Arab patients. The male predominance (77%) and relatively young median age of 48 years suggest that severe bleeding in AML is not confined to the elderly and may affect middle-aged individuals undergoing intensive therapy. While 39% of bleeding patients had at least one significant comorbidity, the majority had no major underlying illness, indicating that bleeding risk in AML is not solely driven by baseline medical fragility. Among the identified subtypes, monocytic AML and AML with myelodysplasia-related changes were in smaller proportions, and only one patient had therapy-related AML. Cytogenetic profiles were frequently diploid, but abnormalities such as MLL rearrangements and del(5q) were also observed. FLT3-ITD and NPM1 mutations were identified in a subset of patients. However, the study's small sample size and retrospective design limit the ability to draw definitive conclusions regarding the association between genetic features and bleeding risk.

The bleeding events were overwhelmingly intracranial (86%), with intracerebral hemorrhage being the most frequent subtype. These events were almost uniformly symptomatic, with 80% of patients presenting with neurological signs such as altered consciousness, anisocoria, or seizures, prompting neuroimaging. Non-neurological bleeding was rare but included serious sites such as the gastrointestinal tract, retroperitoneum, and urinary bladder. Importantly, 40% of bleeding episodes occurred during or shortly after intensive chemotherapy—especially “3 + 7” or HiDAC-based regimens—underscoring the profound impact of treatment-induced cytopenias and coagulopathy. Seventeen percent of events occurred during less-intensive therapies like azacitidine or gilteritinib, while 11% (n = 4) presented before any treatment, possibly reflecting disease-associated hemostatic dysfunction at diagnosis.

Taken together, these findings show that severe bleeding in AML often arises at the intersection of active disease biology and treatment-induced marrow suppression. Laboratory results during bleeding episodes revealed profound cytopenias: thrombocytopenia (median PLT: 11 × 10⁹/L) and anemia (median Hb: 7.1 g/dL) were universal, with frequent leukopenia (median WBC: 1.3 × 10⁹/L) and neutropenia (median ANC: 0.1 × 10⁹/L). This pattern supports the concept that marrow suppression—whether due to disease or therapy—is a central driver of bleeding risk.^12^ In a few cases, hyperleukocytosis was observed, with white blood cell counts exceeding 50-100 × 10⁹/L, which may have contributed to endothelial injury and intracranial bleeding.^14^ Only 6% of patients underwent neurosurgical intervention, emphasizing the limited feasibility of surgical management in this fragile population.

Outcomes after severe bleeding were poor in our cohort, with 41.2% mortality within 30 days and 76.5% overall. Intracranial hemorrhage and septic shock were the leading causes of death, each accounting for roughly one-third of fatalities, while survivors generally had less extensive bleeding or bled outside the induction phase when hematologic recovery was more likely. These findings highlight the early treatment phase as a “perfect storm” of risk, driven by active disease, chemotherapy-induced cytopenia, and infection.^12^ The predominance of intracranial hemorrhage (ICH) underscores the need for proactive, individualized transfusion strategies. Although current guidelines recommend maintaining platelet counts above 10 × 10⁹/L in stable AML and 20-50 × 10⁹/L in high-risk settings, most ICH events occurred below these thresholds (median 11 × 10⁹/L), suggesting that existing practices may not always prevent catastrophic bleeding. Beyond thrombocytopenia, qualitative platelet dysfunction, coagulopathy, and disseminated intravascular coagulation (DIC) may further amplify bleeding risk.^15,16^ Although intracranial hemorrhage accounted for 9 of 26 deaths, most patients had biologically higher-risk AML, often presenting with early bleeding, hyperleukocytosis, or refractory disease, predisposing them to sepsis, DIC, and marrow failure. These observations suggest that the excess mortality reflects a multifactorial process rather than bleeding alone. While racial or genetic factors cannot be entirely excluded, the elevated mortality more plausibly reflects the combined effects of aggressive leukemia biology, disease refractoriness, and treatment-related cytopenia. These findings are consistent with prior reports showing 50%-80% mortality after major hemorrhage in AML^3,12,13,17^ and emphasize the need for early identification of biologically high-risk patients, risk-adapted transfusion thresholds, and intensified supportive care during induction.

When compared with other international cohorts, the incidence and predictors of major bleeding in AML remain similar across regions, with reported rates of severe hemorrhage ranging from 5% to 20% in Western populations.^3–5,17^ However, data from non-Western populations are limited. Our study provides one of the first regional analyses from the Middle East, reflecting a multiethnic population and real-world supportive care practices. By documenting the timing, sites, and outcomes of severe bleeding, our findings extend global knowledge and underscore the need for context-specific prevention strategies.

Finally, several limitations must be acknowledged. The retrospective design introduces potential for selection bias and incomplete data, particularly regarding bleeding extent, timing, and molecular characterization. The sample size, though clinically meaningful, limits statistical power and precludes multivariate analysis. Furthermore, since only patients with Grade ≥ 3 bleeding were analyzed, comparative survival analyses against non-bleeders were not feasible.

## Conclusion

Severe bleeding—especially intracranial hemorrhage—is a common and often fatal complication in AML even among younger patients, typically occurring early in the treatment course. Profound cytopenias, intensive chemotherapy, and disease-associated coagulopathies contribute significantly to bleeding risk. Strengthening supportive-care measures, refining bleeding-risk stratification, and implementing targeted preventive strategies—such as individualised transfusion thresholds and early intervention in high-risk patients—are essential to reduce hemorrhage-related mortality and improve overall treatment outcomes.
